# Associate Lesions of the First Dentition

**Published:** 1898-10

**Authors:** Harry L. Belcher

**Affiliations:** Buffalo, N. Y.


### ASSOCIATE LESIONS OF THE FIRST DENTITION.[1]

BY HARRY L. BELCHER, D.D.S., BUFFALO, N. Y.

It is not my purpose, in presenting this paper, to go into dental evolution, but to take up a few pathological conditions, and touch upon some things which may possibly be of benefit to us in every-day practice.

The process of dentition, while a physiological one, is liable to be one of continuous irritation. It is not, however, by any means understood that all infantile diseases are influenced by, or associated with, dentition. Sometimes infants are tortured, and in many cases have an existing disease aggravated, by lancing and cutting. Dental irritation is influenced by the slowness or rapidity of the evolution of the teeth, by the state of health of the infant, and by idiosyncrasy. We would not anticipate from the eruption of a single tooth the trouble of five or six. Some children erupt their

---

[1] Read before the Barrettonian Society and Buffalo Dental Association.

teeth in mass, while in the case of others it is a process of regularity and harmony. According to Dr. A. Brothers, dentition is rarely, if ever, a direct cause of death. The calculations of Dr. Arbuthnot are that, at the period of dentition, one child in every ten has its life destroyed through the associated and influenced lesions of that age.

Retarded dentition may occur in healthy children, or in entire families.

The period of eruption of the first teeth begins in healthy children at about six and a half months, and is usually complete at thirty months.

Dentition is retarded if the child is not properly fed. The quality of the first teeth depends largely on the physical qualities of the child, and on the ability of the mother to properly nourish the infant.

Congenital diseases—tuberculosis, syphilis, etc.—seem to have a retarding effect on dentition. Scrofulosis seems to hasten the eruption of the first teeth, but does not affect the later teeth.

In cases of undeveloped brain—idiocy—there is a marked retardation during the entire period of dentition.

Chronic diseases have a retarding power over the first teeth, but do not seem to influence the later teeth.

Premature dentition is attended with more danger than later ones. Peabody says, "The later the teeth erupt the stronger and better they will be."

"An abnormal dentition" is indicated by a dry, hot month, "swollen gums, tense, tender, and shining."

The diseases, if they may be so called, associated with and dependent on abnormal dentition are localized stomatitis, irritative fever, diarrhœa, spasms, eruptions upon skin, especially of scalp and face.

The first indication of the condition dependent on advancing eruption consists in a sense of titillation, or itching, before any local sign is visible. The child is found disposed to rub the parts with anything coming into its hands, seeming most comfortable when biting upon a hard substance. Slavering is also associated with this stage. After a time tumefaction of the gums is observed.

Garretson says, "The shape and extent of face in the erupting tooth do not seem to have as much to do with the amount of irritation as one would naturally infer would be the case." He has had as much trouble from an erupting incisor as in the case of a four-cuspid molar. Bad and degenerating inflammations are always associated with constitutional conditions. In scrofulous children it is sometimes the case that a semi-gangrenous ulceration is the result of cutting a tooth,—which is quite troublesome to manage,—while in children of a mercurio-syphilitic cachexia such a condition will be even aggravated, the gums and continuity of mucous membrane looking as if it were impossible to keep the parts from breaking down into general ulceration.

According to some authorities Dr. Barrett concluded the diseases of childhood not to be due to, or dependent on, dentition. He says, "It comes from bad feeding (digestive disturbances), and not from the teeth." He clinches his argument with data to show the death-rate of children under three years of age, and the prevalence of zymotic diseases in certain wards of our own city, where sewerage is poor. He says, further, that there are no real disturbances of dentition unless it shows itself in the gums.

Too much stress cannot be laid on the diet. The stomach of an infant is very weak; can hardly be called a stomach. It may be likened to an enlarged portion of a common tube. Again, the mucous membrane of the intestines of an infant are tender and susceptible. Excess of food, or food not easy of digestion, irritates and excites these tissues, causing discharge or diarrhœa. Thus we see the necessity of a careful diet, and the adoption of hygienic measures.

LANCING.

In regard to lancing, the experienced eye of the operator will determine when this is necessary. Too early lancing is an evil, as the tissues heal and become hardened, calloused as it were, and make it more difficult for the teeth to erupt. When tumefaction of the gum is dependent on tooth eruption, and the child is in healthy condition, a certain evidence is found in the glistening character of the swelling, the part immediately over the tooth looking stretched and feverish. This tense look is nearly always present, and may under all circumstances be taken as an indication demanding the use of the lancet. Lancing in these cases affords almost instant relief. Personally, I do not think the lancet is used effectually. A tooth is to be lanced in consideration of its shape.

A single incision is all that is necessary with upper or lower front teeth. The incision should be made sufficiently deep to feel the lancet strike upon the enamel, being made on a line with the cutting edge of the tooth. With the posterior teeth, the cuspidati included, the crucial form of incision is demanded. This form will relieve the advancing cusps and afford the result desired.

A sharp or razor-edged lancet is preferred by most operators, though the theory has been promulgated that a saw-edge lancet is to be prefered, as it tangles up the vessels, and thus is conducive to the formation of a clot. There certainly is less bleeding when this method is employed. It might be used to advantage on infants whose parents have a tendency to hemorrhage, though perhaps the bleeding that follows most lancing, increased as it is by the child's sucking, encouraged by the saline taste of blood, is advantageous in reducing inflamation.

### THE CARE OF CHILDREN'S TEETH.

Some dentists dread to work on children's teeth. This is a mistake, and we must overcome it, for there is much at stake. This is one of the ways a practice can be built up. One of our most successful practitioners tells me that he secured some of his most influential patients by working for children, gaining the confidence of the little ones by his gentle procedure, and what is more, the confidence of the parents. We know the importance of preserving the temporary teeth as long as possible, for they nourish the permanent teeth, preserve the shape of the arch, prevent irregularities, etc.

A word from the dentist to the child has much more effect than from parents. Tell the child to use a tooth-brush carefully and with regularity. Note if they follow your suggestion. If so, remark on their improved appearance, or again speak more decidely if you notice no improvement.

If possible, exclude parents from operating-room. They spoil authority. The child is not to be controlled, but looks to the parent for sympathy, and will not make an effort to control himself. After the dentist has obtained the confidence of the child he can do most anything, so long as he does not deceive.

I know of no suitable material with which to fill the roots ot temporary teeth. As they must be absorbed, they cannot be filled with an insoluble compound.

In cases of exposure of the pulp, aristol makes an excellent soothing application. Oil of cloves and tannic acid are also recommended. Dr. Southwick suggests the use of a very minute quantity of arsenic in conjunction with carbolic acid.

We have to be satisfied with work that we would be heartily ashamed of in an adult, as it must be done painlessly and quickly. A child will not bear fatigue, and refuses to be hurt.

Though the pulp is much less sensitive in a child's tooth than in an adult's, it is much larger in proportion. One-third of a temporary tooth is taken up by the pulp. The pulp lies much nearer the surface, so great care must be taken not to expose it, though we can resort to capping with a greater expectation of success than with an adult.

Copper amalgam, if not abused in its use, makes an excellent filling for temporary teeth. The salts of copper having a stimulating effect on tooth-structure and pulp. It is especially valuable in fragile teeth. By some operators copper amalgam has been used indiscriminately and excessively, since which time it has received their condemnation. Notwithstanding this, it is a very valuable agent for the preservation of soft teeth, when used intelligently.

Nitrate of silver is a valuable agent to arrest superficial decay of children's teeth. This is especially valuable in early decay; it acts as a check; stimulates the tooth, and encourages secondary deposits. It is used in stick form, and as a solution. Very slight amounts can be used. This agent is rubbed over the decayed part, the tooth being first thoroughly dried. (I dry around it so that the silver will not be distributed through the saliva.)

In closing I will quote Dr. Allport: "The great requisite, as has been said, is to gain the child's confidence. I address myself to the child when I make an appointment, and not to the parent. I say, 'When can you come?' thus giving the little patient an impression of a confidential arrangement between himself and me. This often has a favorable effect upon the child, for children appreciate anything that looks like deference to their wishes, and they will usually meet you half-way when you treat them as principals, where they might be difficult of management if you made some mysterious arrangement with the parent, only half understood by them. The secret of handling our little patients successfully is to do but little at any time, avoiding anything like an air of mystery, and always dealing in perfect candor and honesty of intention with them."

